# 1,5-Diamino-2,6-dibromo-9,10-anthraquinone

**DOI:** 10.1107/S1600536812004229

**Published:** 2012-02-24

**Authors:** Nadine Seidel, Wilhelm Seichter, Edwin Weber

**Affiliations:** a Institut für Organische Chemie, TU Bergakademie Freiberg, Leipziger Strasse 29, D-09596 Freiberg/Sachsen, Germany

## Abstract

In the title compound, C_14_H_8_Br_2_N_2_O_2_, the mol­ecular structure features intra­molecular N—H⋯O [2.639 (2) Å and 130°] and N—H⋯Br [3.053 (2) Å and 114°] hydrogen bonding. Due to inversion symmetry, the asymmetric part of the unit cell consits of one half-mol­ecule. In the crystal, inversion dimers linked by pairs of N—H⋯O [2.955 (2) Å and 135°] hydrogen bonds occur. The structure also features C=O⋯π [3.228 (2) Å] and Br⋯Br [3.569 (1) Å] contacts.

## Related literature

For background information on anthraquinones and their pharmacological potential, see: Thomson (1967 ▶); Bohacova *et al.* (1998 ▶). For the X-ray structure of anthraquinone at different temperatures, see: Fu & Brock (1998 ▶). For a description of the resonance assisted hydrogen bond (RAHB) model, see: Gilli *et al.* (1989 ▶); Sanz *et al.* (2008 ▶). For structures with typical intramolecular N—H⋯Br, N—H⋯O and C=O⋯π contacts, see: Brammer *et al.* (2001 ▶); Shimpi *et al.* (2007 ▶); Marten *et al.* (2007 ▶). For the synthetic procedure, see: Scholl & Krieger (1904 ▶).
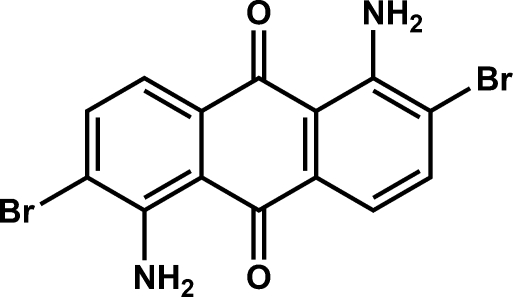


## Experimental

### 

#### Crystal data

C_14_H_8_Br_2_N_2_O_2_
*M* *_r_* = 396.04
Triclinic, 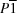

*a* = 4.4177 (1) Å
*b* = 6.2240 (2) Å
*c* = 11.8410 (3) Å
α = 94.455 (2)°
β = 99.970 (2)°
γ = 100.859 (2)°
*V* = 312.87 (2) Å^3^
*Z* = 1
Mo *K*α radiation
μ = 6.48 mm^−1^
*T* = 153 K
0.58 × 0.22 × 0.05 mm

#### Data collection

Bruker APEXII CCD area-detector diffractometer
Absorption correction: multi-scan (*SADABS*; Bruker, 2007 ▶) *T*_min_ = 0.117, *T*_max_ = 0.759
7769 measured reflections
2003 independent reflections
1844 reflections with *I* > 2σ(*I*)
*R*_int_ = 0.029

#### Refinement

*R*[*F* ^2^ > 2σ(*F* ^2^)] = 0.026
*wR*(*F* ^2^) = 0.071
*S* = 0.96
2003 reflections
91 parameters
H-atom parameters constrained
Δρ_max_ = 0.86 e Å^−3^
Δρ_min_ = −0.63 e Å^−3^

### 

Data collection: *APEX2* (Bruker, 2007 ▶); cell refinement: *SAINT* (Bruker, 2007 ▶); data reduction: *SAINT*; program(s) used to solve structure: *SHELXS97* (Sheldrick, 2008 ▶); program(s) used to refine structure: *SHELXL97* (Sheldrick, 2008 ▶); molecular graphics: *ORTEP-3* (Farrugia, 1997 ▶); software used to prepare material for publication: *SHELXTL* (Sheldrick, 2008 ▶).

## Supplementary Material

Crystal structure: contains datablock(s) I, global. DOI: 10.1107/S1600536812004229/im2352sup1.cif

Structure factors: contains datablock(s) I. DOI: 10.1107/S1600536812004229/im2352Isup2.hkl

Supplementary material file. DOI: 10.1107/S1600536812004229/im2352Isup3.cml

Additional supplementary materials:  crystallographic information; 3D view; checkCIF report

## Figures and Tables

**Table 1:** Hydrogen-bond geometry (Å, °)

*D*—H⋯*A*	*D*—H	H⋯*A*	*D*⋯*A*	*D*—H⋯*A*
N1—H1*B*⋯O1	0.88	1.99	2.639 (2)	130
N1—H1*A*⋯Br1	0.88	2.59	3.053 (2)	114
N1—H1*B*⋯O1^i^	0.88	2.27	2.955 (2)	135

Symmetry code: (i) 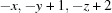.

**Fig. 1.**
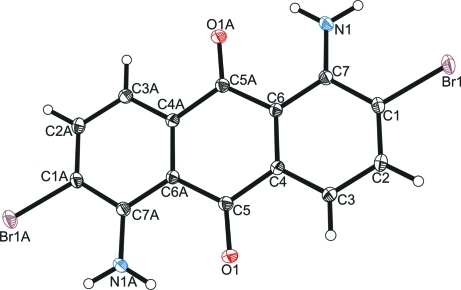
Perspective view of the title compound, showing 50% probability displacement ellipsoids for non-H atoms.

**Fig. 2.**
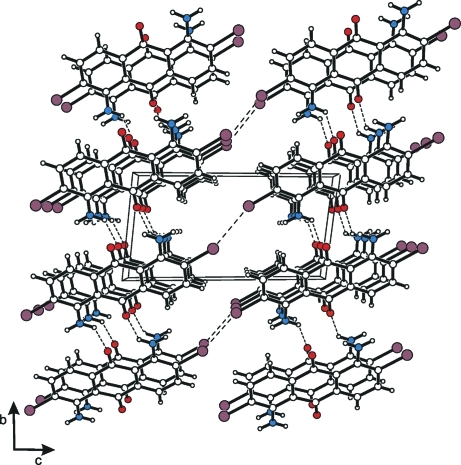
Packing diagram of the title compound viewed down the a axis. Intermolecular contacts are shown as dashed lines.

## supplementary crystallographic information

### Comment

Anthraquinones represent the most important group of natural quinones (Thomson, 1967). They also find extensive application in chemical research and industry including aspects of pharmacology (Bohacova *et al.*, 1998). The title compound crystallizes in the triclinic space group *P*1 with one half of the molecule in the asymmetric unit of the unit cell, i.e. the molecule adopts inversion symmetry. The anthraquinone part shows an almost planar geometry except for the bromo atoms which are slightly twisted out of the plane (*Cg*(1)—C(1)—Br(1) 178.0 (2) °). Compared with the unsubstituted anthraquinone, the position of atoms shows distinct differences. The C—C bond lengths [C(6)—C(7) 1.421 (2) Å, C(1)—C(7) 1.420 (2) Å] are considerably elongated compared with 1.393 (2) Å, 1.381 (2) Å for anthraquinone. On the other hand, the angle of C(6)—C(7)—C(1) 116.5 (2) ° is significantly reduced compared with 120.39 (14) ° for anthraquinone (Fu & Brock, 1998). This can be explained by the so called 'resonance assisted hydrogen bond (RAHB) model', which is based on the assumption of a synergistic mutual reinforcement of intramolecular hydrogen bonding due to the conjugation of π electrons (Gilli *et al.*, 1989). In our case, the amino group of the molecule seems to be involved in an intramolecular hydrogen bond of this type [N1—H1B···O1 2.639 (2) Å, 130 °]. However, this particular conception is also disputed (Sanz *et al.*, 2008). The amino group is also involved in an intramolecular N—H···Br contact [N1—H1A···Br1 3.053 (2) Å, 114 °] (Brammer *et al.*, 2001). In addition, intermolecular hydrogen bonds of the N—H···O type are found [N1—H1B···O1 2.955 (3) Å, 135 °]. In with way tapes are generated, which are further cross-linked by Br···Br-contacts [Br···Br 3.569 (1) Å, 155.8 (1) °] (Shimpi *et al.*, 2007). The resulting two-dimensional networks are stacked *via* C═O···π interactions [C(5)···*Cg*(2) 3.228 (2) Å, 97.28 (13) °] (Marten *et al.*, 2007).

### Experimental

The title compound was synthesized by bromination of 1,5-diaminoanthra-9,10-quinone (1.0 g, 4.20 mmol) with bromine in acetic acid. For the synthetic procedure, see: Scholl & Krieger (1904). Purification by column chromatography (silica gel, *n*-hexane:ethyl acetate/4:1) followed by crystallization from toluene yielded 1.1 g (68%) of the compound as red prismatic crystals.

### Refinement

H atoms were positioned geometrically and allowed to ride on their respective parent atoms, with C—H = 0.95 Å and *U*_iso_(H) = 1.2*U*_eq_(C) for aryl and N—H = 0.88 Å and *U*_iso_(H) = 1.2*U*_eq_(N) for amino atoms.

### Crystal data

**Table:**

C_14_H_8_Br_2_N_2_O_2_	*Z* = 1
*M**_r_* = 396.04	*F*(000) = 192
Triclinic, *P*1	*D*_x_ = 2.102 Mg m^−^^3^
Hall symbol: -P 1	Mo *K*α radiation, λ = 0.71073 Å
*a* = 4.4177 (1) Å	Cell parameters from 5166 reflections
*b* = 6.2240 (2) Å	θ = 3.2–38.8°
*c* = 11.8410 (3) Å	µ = 6.48 mm^−^^1^
α = 94.455 (2)°	*T* = 153 K
β = 99.970 (2)°	Irregular, red
γ = 100.859 (2)°	0.58 × 0.22 × 0.05 mm
*V* = 312.87 (2) Å^3^	

### Data collection

**Table:**

Bruker APEXII CCD area-detector diffractometer	2003 independent reflections
Radiation source: fine-focus sealed tube	1844 reflections with *I* > 2σ(*I*)
Graphite monochromator	*R*_int_ = 0.029
φ and ω scans	θ_max_ = 31.1°, θ_min_ = 3.4°
Absorption correction: multi-scan (*SADABS*; Bruker, 2007)	*h* = −6→6
*T*_min_ = 0.117, *T*_max_ = 0.759	*k* = −9→9
7769 measured reflections	*l* = −17→17

### Refinement

**Table:**

Refinement on *F*^2^	Primary atom site location: structure-invariant direct methods
Least-squares matrix: full	Secondary atom site location: difference Fourier map
*R*[*F*^2^ > 2σ(*F*^2^)] = 0.026	Hydrogen site location: inferred from neighbouring sites
*wR*(*F*^2^) = 0.071	H-atom parameters constrained
*S* = 0.96	*w* = 1/[σ^2^(*F*_o_^2^) + (0.0454*P*)^2^ + 0.2515*P*] where *P* = (*F*_o_^2^ + 2*F*_c_^2^)/3
2003 reflections	(Δ/σ)_max_ < 0.001
91 parameters	Δρ_max_ = 0.86 e Å^−^^3^
0 restraints	Δρ_min_ = −0.63 e Å^−^^3^

### Special details

**Table:**

Geometry. All e.s.d.'s (except the e.s.d. in the dihedral angle between two l.s. planes) are estimated using the full covariance matrix. The cell e.s.d.'s are taken into account individually in the estimation of e.s.d.'s in distances, angles and torsion angles; correlations between e.s.d.'s in cell parameters are only used when they are defined by crystal symmetry. An approximate (isotropic) treatment of cell e.s.d.'s is used for estimating e.s.d.'s involving l.s. planes.
Refinement. Refinement of *F*^2^ against ALL reflections. The weighted *R*-factor *wR* and goodness of fit *S* are based on *F*^2^, conventional *R*-factors *R* are based on *F*, with *F* set to zero for negative *F*^2^. The threshold expression of *F*^2^ > σ(*F*^2^) is used only for calculating *R*-factors(gt) *etc*. and is not relevant to the choice of reflections for refinement. *R*-factors based on *F*^2^ are statistically about twice as large as those based on *F*, and *R*-factors based on ALL data will be even larger.

### Fractional atomic coordinates and isotropic or equivalent isotropic displacement parameters (Å^2^)

**Table:**

	*x*	*y*	*z*	*U*_iso_*/*U*_eq_	
Br1	0.18844 (5)	0.30597 (3)	0.572710 (15)	0.02812 (8)	
O1	0.1853 (3)	0.3189 (2)	1.04118 (13)	0.0228 (3)	
N1	0.1114 (4)	0.3920 (3)	0.82251 (13)	0.0197 (3)	
H1A	0.0439	0.4563	0.7622	0.024*	
H1B	0.0777	0.4351	0.8910	0.024*	
C1	0.3215 (4)	0.1566 (3)	0.69978 (14)	0.0184 (3)	
C2	0.4697 (4)	−0.0157 (3)	0.68165 (15)	0.0205 (3)	
H2	0.4972	−0.0606	0.6062	0.025*	
C3	0.5785 (4)	−0.1235 (3)	0.77398 (15)	0.0188 (3)	
H3	0.6811	−0.2421	0.7618	0.023*	
C4	0.5371 (4)	−0.0574 (3)	0.88430 (14)	0.0152 (3)	
C5	0.3340 (4)	0.1755 (3)	1.02023 (15)	0.0159 (3)	
C6	0.3806 (4)	0.1154 (3)	0.90319 (14)	0.0145 (3)	
C7	0.2662 (4)	0.2273 (3)	0.80995 (14)	0.0158 (3)	

### Atomic displacement parameters (Å^2^)

**Table:**

	*U*^11^	*U*^22^	*U*^33^	*U*^12^	*U*^13^	*U*^23^
Br1	0.04356 (14)	0.03062 (12)	0.01527 (11)	0.01733 (9)	0.00600 (8)	0.00918 (7)
O1	0.0304 (7)	0.0252 (7)	0.0177 (6)	0.0166 (5)	0.0062 (5)	0.0020 (5)
N1	0.0264 (7)	0.0191 (7)	0.0164 (7)	0.0110 (6)	0.0032 (5)	0.0048 (5)
C1	0.0223 (7)	0.0205 (8)	0.0132 (7)	0.0063 (6)	0.0027 (5)	0.0042 (6)
C2	0.0257 (8)	0.0246 (8)	0.0123 (7)	0.0074 (6)	0.0045 (6)	0.0019 (6)
C3	0.0249 (8)	0.0185 (7)	0.0152 (7)	0.0090 (6)	0.0050 (6)	0.0011 (6)
C4	0.0161 (7)	0.0162 (7)	0.0138 (7)	0.0044 (5)	0.0030 (5)	0.0012 (5)
C5	0.0167 (7)	0.0154 (7)	0.0156 (7)	0.0041 (5)	0.0027 (5)	0.0016 (6)
C6	0.0161 (7)	0.0150 (7)	0.0127 (7)	0.0038 (5)	0.0026 (5)	0.0011 (5)
C7	0.0173 (7)	0.0154 (7)	0.0150 (7)	0.0038 (5)	0.0027 (5)	0.0019 (5)

### Geometric parameters (Å, º)

**Table:**

Br1—C1	1.8949 (18)	C2—H2	0.9500
O1—C5	1.238 (2)	C3—C4	1.392 (2)
N1—C7	1.348 (2)	C3—H3	0.9500
N1—H1A	0.8800	C4—C6	1.407 (2)
N1—H1B	0.8800	C5—C6	1.467 (2)
C1—C2	1.379 (3)	C5—C4^i^	1.485 (2)
C1—C7	1.420 (2)	C6—C7	1.421 (2)
C2—C3	1.389 (3)		
			
C7—N1—H1A	120.0	C3—C4—C6	120.66 (16)
C7—N1—H1B	120.0	C3—C4—C5^i^	117.24 (15)
H1A—N1—H1B	120.0	C6—C4—C5^i^	122.10 (15)
C2—C1—C7	122.64 (16)	O1—C5—C6	121.83 (16)
C2—C1—Br1	118.66 (13)	O1—C5—C4^i^	119.18 (16)
C7—C1—Br1	118.69 (13)	C6—C5—C4^i^	118.98 (15)
C1—C2—C3	119.84 (16)	C4—C6—C7	120.42 (15)
C1—C2—H2	120.1	C4—C6—C5	118.89 (15)
C3—C2—H2	120.1	C7—C6—C5	120.69 (15)
C2—C3—C4	119.87 (16)	N1—C7—C1	120.38 (16)
C2—C3—H3	120.1	N1—C7—C6	123.08 (15)
C4—C3—H3	120.1	C1—C7—C6	116.54 (15)
			
Br1—Br1—C1—C2	0.00 (9)	O1—C5—C6—C4	−176.53 (16)
Br1—Br1—C1—C7	0.00 (10)	C4^i^—C5—C6—C4	2.1 (2)
C7—C1—C2—C3	−1.7 (3)	O1—C5—C6—C7	2.6 (2)
Br1—C1—C2—C3	177.95 (14)	C4^i^—C5—C6—C7	−178.77 (14)
C1—C2—C3—C4	0.1 (3)	C2—C1—C7—N1	−177.84 (17)
C2—C3—C4—C6	1.1 (3)	Br1—C1—C7—N1	2.5 (2)
C2—C3—C4—C5^i^	−178.47 (16)	C2—C1—C7—C6	1.9 (2)
O1—O1—C5—C6	0.0 (5)	Br1—C1—C7—C6	−177.76 (12)
O1—O1—C5—C4^i^	0.0 (6)	C4—C6—C7—N1	179.15 (16)
C3—C4—C6—C7	−0.9 (2)	C5—C6—C7—N1	0.0 (2)
C5^i^—C4—C6—C7	178.70 (15)	C4—C6—C7—C1	−0.6 (2)
C3—C4—C6—C5	178.25 (15)	C5—C6—C7—C1	−179.71 (15)
C5^i^—C4—C6—C5	−2.2 (3)		

Symmetry code: (i) −*x*+1, −*y*, −*z*+2.

### Hydrogen-bond geometry (Å, º)

**Table:**

*D*—H···*A*	*D*—H	H···*A*	*D*···*A*	*D*—H···*A*
N1—H1*B*···O1	0.88	1.99	2.639 (2)	130
N1—H1*A*···Br1	0.88	2.59	3.053 (2)	114
N1—H1*B*···O1^ii^	0.88	2.27	2.955 (2)	135

Symmetry code: (ii) −*x*, −*y*+1, −*z*+2.
